# Induction of premature senescence and a less-fibrogenic phenotype by programmed cell death 4 knockdown in the human hepatic stellate cell line Lieming Xu-2

**DOI:** 10.1007/s13577-022-00844-9

**Published:** 2022-12-16

**Authors:** Rasheda Perveen, Iwata Ozaki, M. Manirujjaman, Keiichiro Mine, Yuzo Murata, Kenichi Tanaka, Jinghe Xia, Hirokazu Takahashi, Keizo Anzai, Sachiko Matsuhashi

**Affiliations:** 1 Division of Hepatology, Diabetology and Endocrinology, Department of Internal Medicine, Saga Medical School, Saga University, 5-1-1 Nabeshima, Saga, Saga 849-8501 Japan; 2 Health Administration Center, Saga Medical School, Saga University, 5-1-1 Nabeshima, Saga, Saga 849-8501 Japan; 3 Department of Pharmaceutical Sciences, School of Pharmacy at Fukuoka, International University of Health and Welfare, 137-1 Enokizu, Ohkawa, Fukuoka 831-8501 Japan; 4 Liver Disease Center, Saga University Hospital, Saga Medical School, Saga University, 5-1-1 Nabeshima, Saga, Saga 849-8501 Japan

**Keywords:** PDCD4, p21, Hepatic stellate cell, α-SMA, Senescence, Fibrosis

## Abstract

Although programmed cell death 4 (PDCD4) was initially reported as a tumor suppressor and has been shown to inhibit cancer cell growth and metastasis, recent studies have demonstrated that loss of PDCD4 expression also induces growth inhibition by inducing apoptosis and/or cellular senescence. At present, the roles of PDCD4 in the activation and profibrogenic properties of myofibroblasts, which are critically involved in organ fibrosis, such as that in the liver, are unclear. We, therefore, investigated the roles of PDCD4 in myofibroblasts using human hepatic stellate cell line Lieming Xu-2 (LX-2). PDCD4 knockdown inhibited LX-2 proliferation and induced a senescent phenotype with increased β-galactosidase staining and p21 expression in a p53-independent manner together with downregulation of the notch signaling mediator RBJ-κ/CSL. During PDCD4 knockdown, alpha smooth muscle actin (α-SMA; an activation marker of myofibroblasts), matrix metalloproteinases MMP-1 and MMP-9, and collagen IV were upregulated, but the expression of collagen1α1 and collagen III was markedly downregulated without any marked change in the expression of tissue inhibitor of metalloproteinase-1 (TIMP-1). These results demonstrated that knockdown of PDCD4 induced the cellular senescence phenotype and activated myofibroblasts while suppressing the profibrogenic phenotype, suggesting roles of PDCD4 in cellular senescence and fibrogenesis in the liver.

## Introduction

Human hepatic stellate cells (HSCs) account for 5–8% of all liver cells and play important roles in the storage of vitamin A and production of extracellular matrix (ECM) and metalloproteinases [1–3]. During liver injury, HSCs become activated and transdifferentiate into alpha smooth muscle actin (α-SMA)-rich myofibroblasts [4–6], and this transdifferentiation leads to an increased expression of ECM components and profibrogenic mediators, ultimately causing liver fibrosis [7, 8].

While ECM-producing activated myofibroblastic HSCs are usually resistant to cell death via the activation of anti-apoptotic protein expression [9], it has also been reported that activated HSCs returned to an inactive phenotype during liver fibrosis resolution. During regression of liver fibrosis, senescent HSCs are observed, and it has been shown that induction of cellular senescence can reduce fibrosis progression [10–12].

Cellular senescence was first described as limited replicative potential of human normal fibroblasts in culture, a phenomenon termed replicative senescence because of progressive telomerase shortening [13, 14]. Later, it was shown that senescence can be induced by a variety of conditions, such as an aberrant oncogenic activation, DNA damage, oxidative stress, and chemical agents in the absence of telomerase loss or dysfunction, and this type of cellular senescence is called premature senescence [15]. For example, DNA damage is a common cause of various types of senescence mediated by a common signaling pathway in which the upregulation of p21 (CIP1/WAF1) and downregulation of cyclin-dependent kinase (CDK) proteins are observed [16, 17].

Programmed cell death 4 (PDCD4) is initially isolated as a tumor suppressor. PDCD4 is frequently downregulated in many types of cancers, and the loss of its expression has been strongly implicated in the development and progression of several tumors [18–24]. Although previous studies have shown that PDCD4 suppresses cancer cell growth by inducing apoptosis [24], recent studies have found that the inhibition of PDCD4 also induces inhibition of cell growth by inducing apoptosis and/or cellular senescence [25, 26]. We recently reported that PDCD4 knockdown suppressed cell growth by inducing cellular senescence in a p21-dependent manner in human hepatocellular carcinoma (HCC) cells [27].

Currently, the role of PDCD4 in fibrosis is unclear. Since the induction of senescence is reported to limit the fibrotic response in myofibroblasts [11], we investigated the effects of PDCD4 on cell growth, cellular senescence, and profibrogenic properties in myofibroblasts using the human HSC cell line Lieming Xu-2 (LX-2).

## Materials and methods

### Cell cultures

The human HSC line LX-2 was a generous gift from Dr. Scott Friedman (Department of Medicine, Icahn School of Medicine at Mount Sinai, New York, New York). The cells were cultured and maintained in Dulbecco's modified Eagle’s medium (DMEM; Sigma-Aldrich, St. Louis, MO, USA) containing 10% fetal bovine serum (FBS) (Nichirei Bioscience incorporation Ltd., Tokyo, Japan) and 100 µg/ml penicillin and streptomycin in 5% CO_2_ at 37 °C.

### Reagents

For live and dead cell staining, a Zombie Aqua Fixable Viability kit (423101) was purchased from BioLegend (San Diego, CA, USA). eBioscience FOXp3/Transcription factor staining buffer for cell permeabilization and fixation was obtained from Invitrogen (Thermo Fisher Scientific, Waltham, MA, USA). 7-amino-actinomycin D (7-AAD) dye for nuclear staining was purchased from Sony Biotechnology (San Jose, CA, USA). A PMC-Gelatin zymography kit (ATTO type- AK45) was obtained from Cosmo Bio Co., Ltd. (Tokyo, Japan).

### Antibodies

The mouse monoclonal antibodies of PDCD4 (sc-376430), collagen1α1 (sc-293182), MMP-1 (sc-21731), TIMP-1 (sc-365905), and MMP-9 (sc-13520) were obtained from Santa Cruz Biotechnology, Inc. (Dallas, TX, USA). The antibodies against rabbit-collagen1α1 (ab-34710) and α-SMA (ab-5694) were obtained from Abcam (Cambridge, UK). Antibodies against β-actin, Rb, phospho-Rb at Ser 780 (p-Rb[780]), phospho-p53 at Ser15 (p-p53[Ser15]), p53, p21, CDK2, CDK4, CDK6, cyclin D1, RBPJ-κ/CSL and HRP-linked secondary antibodies for mouse and rabbit antibodies were purchased from Cell Signaling Technology (Beverly, MA, USA). Alexa Flour 488 donkey anti-rabbit IgG (H + L) antibody (A-21206) was obtained from Invitrogen (Thermo Fisher Scientific). PE-conjugated anti-human Ki-67 antibody (350503) was purchased from BioLegend. The antibodies were used according to the protocols provided by corresponding companies.

### siRNA-mediated knockdown of PDCD4, p21, and CSL

The cells were used for transfection when they were 80–90% confluent in 100-mm dishes. siRNA transfection was performed using Lipofectamine RNAiMAX (Life Technologies, Rockville, MD, USA) by the reverse transfection method according to the manufacturer’s protocols. The sequences for the PDCD4-specific siRNA p2 [27] and k603 targeted sequence [28] were 5′-GCACAACUGAUGUGGAAAA-3′ and 5′-GUGUUGGCAGUAUCCUUAG-3′ respectively, p21-specific siRNA [27] was 5′-AUAAUUAAGACACACAAACTG-3′, siRNA sequences for CSL were (1) RBPJ2 5′-GCACUCCCAAGAUUGAUAA-3′, (2) RBPJ3 5′-CUGACUCAGACAAGCGAAA-3′ [29] and all of which were prepared by Hokkaido System Science Co. Ltd. (Hokkaido, Japan). The Allstar negative control siRNA (1027281) was obtained from Qiagen (Heiden, Germany). Approximately 2 × 10^5^ LX-2 cells were seeded in 35-mm dishes for continued culturing after treatment with PDCD4 and CSL siRNA via the reverse transfection method. The cells were collected for Western blot and qRT-PCR analysis at 1, 3, and 5 days after transfection.

### Transfection of plasmids

LX-2 cells were transfected with PDCD4 expression plasmids [24] by electroporation using LONZA-2B according to the manufacturer’s instruction. In brief, cells were trypsinized, and 1 × 10^6^ cells were taken in the cuvette and mixed with the plasmid and LONZA kit-T (specific for LX-2 cells). Suspensions were kept at room temperature for 10 min after electroporation. An appropriate amount of media was then added, and cells were seeded into 35-mm dishes for continued culture time as mentioned in the figure.

### Cell growth assays

siRNA transfection was performed using the reverse transfection method for LX-2 cells, as described above. In brief, approximately 2 × 10^4^ LX-2 cells were seeded in 24-well plates for continued culturing after treatment with siRNA via the reverse transfection method. At the culture times indicated in the figures, the cells were trypsinized and suspended with DMEM and 0.4% Trypan Blue. The numbers of viable cells were counted using a hemocytometer as per standard protocol.

### Cell proliferation assays

The proliferation activity of PDCD4 knockdown cells was determined by flow cytometry (FACS) with Ki-67 staining. In brief, after treatment with siRNA, 2 × 10^5^ LX-2 cells were seeded in a 35-mm culture dish. At the culture times indicated in the figures, cells were trypsinized and washed with 1 × phosphate-buffered saline (PBS). After staining with zombie aqua dye, cells were permeabilized, fixed, and stained with Ki-67 antibody according to protocol B for the FoxP3 buffer solution (Thermo Fisher Scientific). After being washed twice, cells were suspended in FACS buffer (1% heat-inactivated fetal calf serum, 1 mM EDTA, 0.05% NaN_3_) and incubated for 5 min with 7-AAD solution. The cells were then analyzed by a flow cytometer (BD FACSVerse™; BD BioSciences, NJ, USA), and the percentages of Ki-67-positive cells were determined.

### The β-galactosidase activity assay

Cellular senescence was assessed using the β-galactosidase staining kit (Cell Signaling) following the manufacturer’s protocols. Cells treated with siRNAs were cultured in 35-mm dishes for 1, 3, and 5 days. After washing with PBS, the cells were fixed and stained for β-galactosidase activity using the staining solution overnight at 37 °C, and counterstained with Kernechtrot (Sigma–Aldrich). Stained cells were observed under a microscope and photographed. The data were expressed as the percentage of β-galactosidase-positive cells.

### Western blot analyses

The collected cells were extracted by sonication in lysis buffer containing 50 mM Tris (pH 6.8), 2.3% sodium dodecyl sulphate (SDS), and 1 mM phenylmethylsulfonyl fluoride (PMSF). The cell debris was eliminated by centrifugation at 12,000 g for 10 min, and the supernatant was collected. Protein amounts were determined with a DC™ protein assay kit (Bio-Rad, Hercules, CA, USA) using bovine serum albumin as the standard by the Lowry method. Protein (10–30 µg) from each sample was mixed with SDS loading buffer, separated by SDS polyacrylamide gel electrophoresis, and transferred to a polyvinylidene difluoride (PVDF) membrane (Bio-Rad). The membrane was blocked via incubation overnight at 4 °C in PBS containing 0.1% Tween 20 and 10% skim milk and then incubated with the primary antibody with shaking for 1 h at room temperature or overnight at 4 °C. After washing 5 times with PBS containing 0.1% Tween 20, the specific bands were visualized by further incubation with horseradish peroxidase (HRP)-conjugated secondary antibody followed by enhanced chemiluminescence detection using the Super Signal™ West Pico PLUS Chemiluminescent Substrate (Thermo Fisher Scientific, Waltham, MA, USA) according to the manufacturer’s instructions. For the detection of p-Rb (780) and p-p53 (Ser15) Tris-buffered saline (TBS) was used instead of PBS. The rabbit polyclonal anti-β-actin antibody was used as a control. The stained membrane was exposed to Fuji Medical X-ray film (Tokyo, Japan), and the specific protein bands were determined with the Image J software program (https://imagej.nih.gov/ij/) and normalized to β-actin.

### Quantitative real-time reverse transcription-polymerase chain reaction (qRT-PCR)

Total RNA was isolated from treated cells using RNAiso Plus (Takara, Kusatsu, Japan) and reverse transcribed to cDNA using a High-Capacity cDNA Reverse Transcription Kit (Thermo Fisher Scientific) according to the manufacturer’s instructions. Real-time PCR using Power-up SYBR Select Master Mix (Thermo Fisher Scientific) was performed on a QuantStudio^TM^3 System (Applied Biosystems; Thermo Fisher Scientific) according to the manufacturer’s instructions. The primers of GAPDH (OriGene qStar-NM-002046), PDCD4 and p21 [27], α-SMA, collagen1α1, collagen III [30], collagen IV [31], MMP-1 (OriGene qStar-NM-002421), MMP-9 [32] and TIMP-1 [33] were synthesized by Hokkaido System Science Co., Ltd. The sequences of primers were as follows: GAPDH forward (F) 5′-GTCTCCTCTGACTTCAACAGCG-3′ and reverse (R) 5′-ACCACCCTGTTGCTGTAGCCAA-3′; PDCD4, F 5′-ATGAGCAGATACTGAATGTAAAC-3′ and R 5′-CTTTACTTCCTCAGTCCCAGCAT-3′; p21, F 5′-TCTTGTACCCTTGTGCCTCG-3′ and R 5′-ATCTGTCATGCTGGTCTGCC-3′; α-SMA, F 5′-GAGACCCTGTTCCAGCCATC-3′ and R 5′-TACATAGTGGTGCCCCCTGA-3′; collagen1α1, F 5′-GGTGACAAGGGTGAGACAGG-3′ and R 5′-GGGACCTTGTTCACCAGGAG-3′; collagen III, F 5′-CTGAACTTCCTGAAGATGTCCTTGA-3′ and R 5′-GCTCGGCTGGAGAGAAGTC-3′; collagen IV, F 5′-ACTACTCGTACTGGCTGTCCA-3′ and R 5′- GGGTACGGTGGGATCTGAATG-3′; MMP-1, F 5′-ATGAAGCAGCCCAGATGTGGAG-3′ and R 5′-TGGTCCACATCTGCTCTTGGCA-3′; MMP-9, F 5′-TTGACAGCGACAAGAAGTGG-3′ and R 5′-GCCATTCACGTCGTCCTTAT-3; TIMP-1, F 5′-AGGTCCCCCTGGAAAGAA-3′ and R 5′-AATCCTCGAGCACCCTGA-3′. Data were analyzed using the standard curve method and the expression of target genes was normalized to GAPDH.

### Immunofluorescence staining

A total of 2 × 10^5^ control and siRNA-treated LX-2 cells were plated onto glass coverslips (Matsunami Glass Co., Osaka, Japan) in 35-mm dishes. After treatment, the cells were washed with 1 × PBS and fixed with 4% paraformaldehyde by incubating for 20 min at room temperature followed by washing thrice with 1 × PBS. The fixed cells were pretreated with 1% bovine serum albumin and 1% donkey serum in PBS for 30 min at room temperature and incubated overnight with α-SMA and collagen1α1 (rabbit) antibody at 4 °C. After washing three times with 1 × PBS, cells were incubated for 1 h with Alexa Fluor 488 donkey anti-rabbit IgG (H + L) as a secondary antibody. 4′,6-Diamidino-2-phenylindole (DAPI) dihydrochloride (Dojindo, Kumamoto, Japan) was used for nuclear staining. Cells were mounted in PermaFluor Mountant (Thermo Fisher Scientific). Stained images were captured using a confocal microscope (LSM880; Carl Zeiss, Oberkochen, Germany) at 20-fold magnification. The Zen software program (Carl Zeiss) was used for image processing.

### Gelatin zymography

Proteolytic activity of MMPs was performed by gelatin zymography of PDCD4 knockdown cell extracts and conditioned medium. At 2 and 3 days after PDCD4 knockdown, the cells were washed, and DMEM without FBS was added, followed by a further 2-day culture. The culture medium was then collected, and cell debris was removed. The cultured medium was concentrated with a Vivaspin protein concentrator-5MWCO (Sartorius Stadim Lab. Ltd., Stonehouse, UK), and the protein amount was determined by a NANODROP 2000c Spectrophotometer (Thermo Fisher Scientific). The same amount of protein as indicated in the figure was then applied. Cell extract was prepared by NP-40 lysis buffer as described by Tajhya et al. [34]. Approximately 15 µg of protein from each sample was used as input, and zymography was performed according to the manufacturer’s protocol. Zymography images were obtained by FUSION FX (Vilber, Marne-la-Vallée, France).

### Statistical analyses

All experiments were performed at least in triplicate. Statistical analyses were performed using Student's *t* test, and *p* < 0.05 was considered to be statistically significant. Results are expressed as the mean ± standard deviation (SD).

## Results

### PDCD4 knockdown induced cell growth suppression and cellular senescent phenotype

**Fig. 1:**
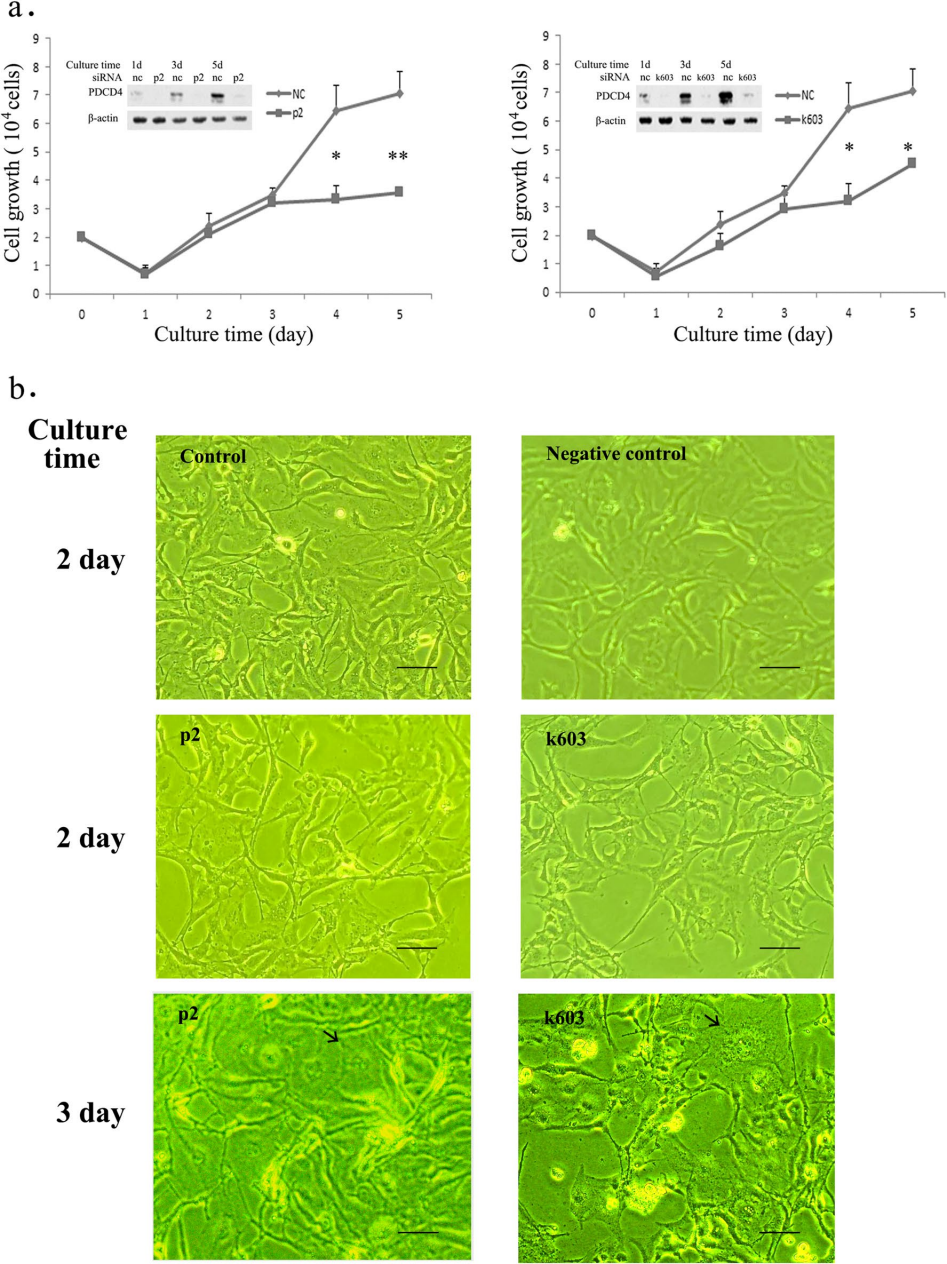
Cell growth of PDCD4 knockdown LX-2 cells. **a** Growth curve of PDCD4 knockdown cells. LX-2 cells were treated with p2 PDCD4-specific and negative control (nc) siRNA (left panel) or with k603 PDCD4-specific and negative control (nc) siRNA (right panel). The data are represented by three individual experiments. The numbers of PDCD4 knockdown LX-2 cells, cultured at days 3 and 5 were significantly lower than those of negative control cells. *t* test, *p < 0.05 and **p < 0.005. **b** Phase-contrast images of no treatment (control), negative control siRNA treated (negative control) at 2 days and PDCD4-specific siRNA-treated (p2 and k603) LX-2 cells at 2 and 3 days after siRNA treatments. Giant cells with cytoplasmic granularity are marked with arrows. Scale bar indicates 100 µm

We first assessed the impact of PDCD4 knockdown on the cell growth of the immortalized LX-2 cells. LX-2 cells were treated with two kinds of PDCD4-specific siRNA (p2 and k603) [27]. As shown in Fig. 1a, the cell numbers in both PDCD4 knockdown and negative control sets were similar initially. The growth of PDCD4 siRNA-treated cells was slightly inhibited compared with that of the negative control cells. After 3 days of knockdown, the growth of PDCD4 knockdown cells had slowed compared to the negative control cells (Fig. 1a). In the PDCD4 knockdown cell cultures, large cells with a flattened shape and cellular granularity in cytoplasm were noted compared to the control and negative control cells, and such cells were increased at 3 days after knockdown (Fig. 1b).

**Fig. 2:**
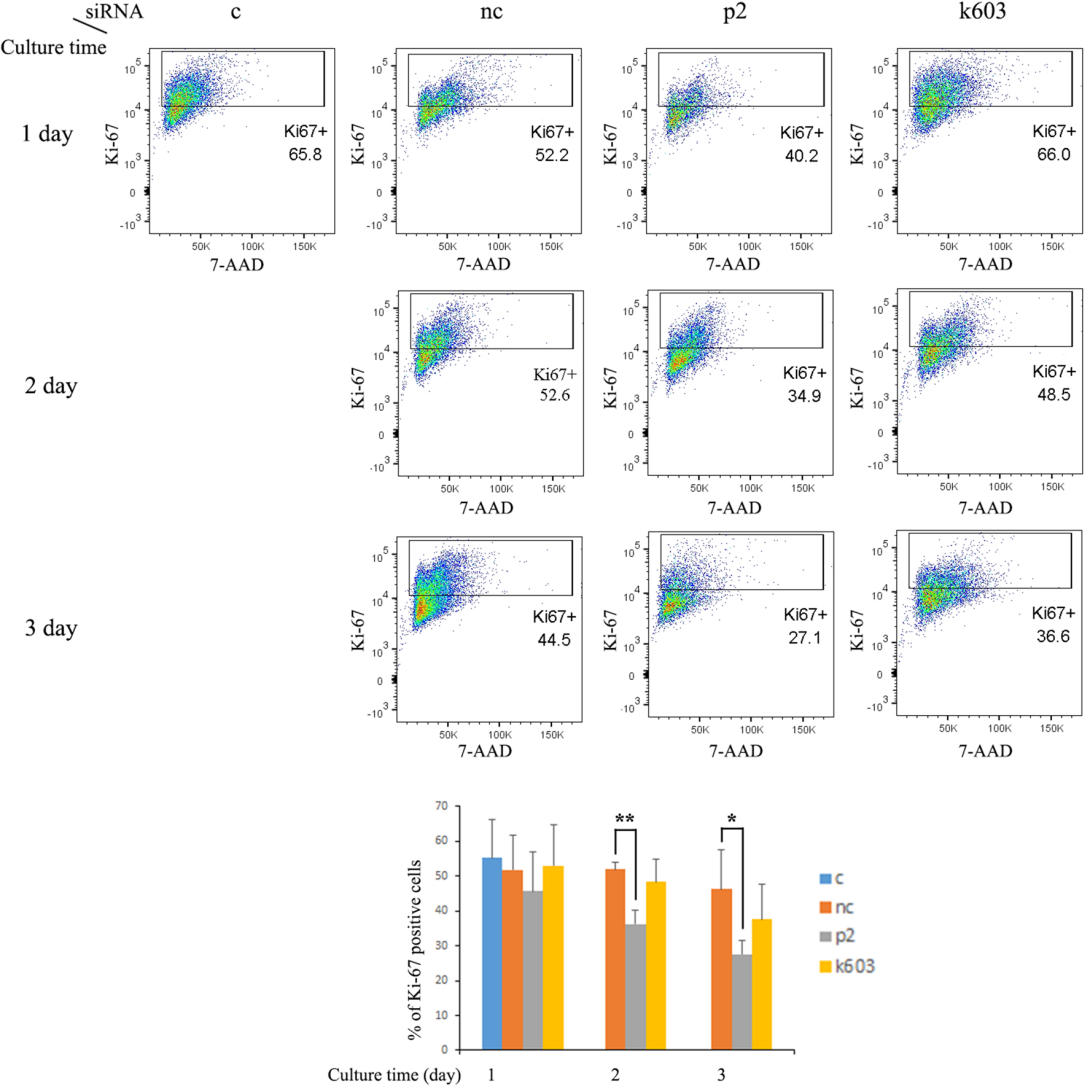
Cell proliferation activity assays of PDCD4 knockdown LX-2 cells. Control cells on day 1 and negative control and PDCD4-specific (p2 and k603) siRNA-treated LX-2 cells on days 1, 2, and 3 were stained with anti-Ki-67 antibody and 7-AAD and analyzed by flow cytometry. After the exclusion of dead and doublet cells, single cells were gated in a combination of Ki-67 and 7-AAD staining. This experiment was repeated four times independently, and the upper panel represents the gate of one experiment. The diagram shows the average percentage of Ki-67-positive cells of the four experiments (lower panel). *t* test, *p < 0.05, **p < 0.005

To assess the mitogenic activity of PDCD4 knockdown cells, the expression of the cell proliferation marker Ki-67 was analyzed by FACS. As shown in Fig. 2, Ki-67-positive cells were decreased in both p2 and k603-siRNA-mediated PDCD4 knockdown cells compared to negative control siRNA-treated cells. However, in the k603-siRNA-mediated PDCD4 knockdown cells, the downregulation of the number of Ki-67-positive cells was not significant.

**Fig. 3:**
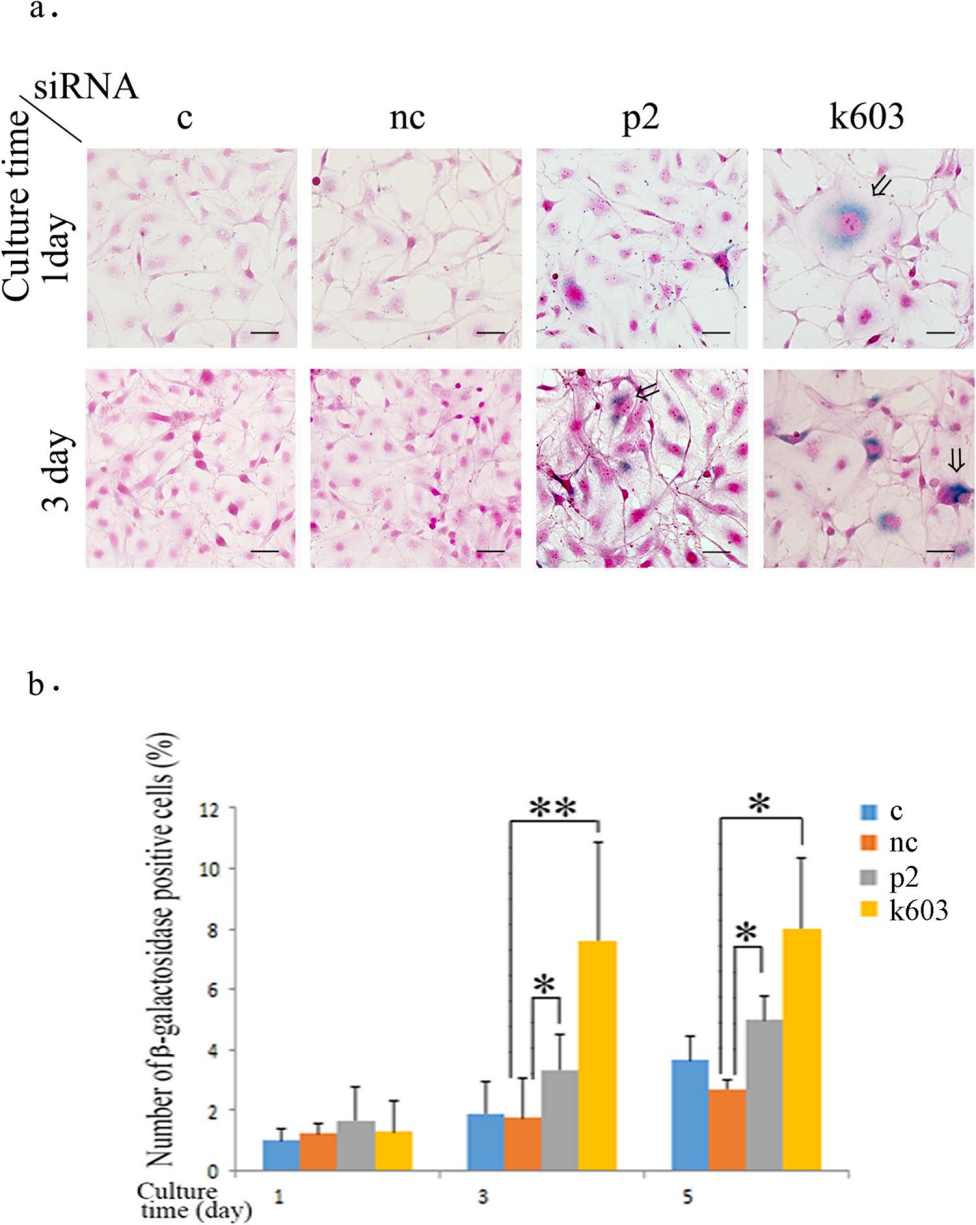
PDCD4 knockdown induced senescence in LX-2 cells. **a** β-Galactosidase staining patterns of control (c), negative control (nc), and PDCD4-specific siRNA (p2 and k603) after treatment at 1 and 3 days. At days 3 and 5, after PDCD4 knockdown, cells with an enlarged, flattened shape, and β-galactosidase positivity were observed. Large nuclei in β-galactosidase-positive cells are marked with arrows. The scale bar indicates 40 µm. **b** The number of β-galactosidase-positive cells. The data were obtained from **a**. The results are depicted from three independent experiments. *t* test, *p < 0.05 and **p < 0.005

To investigate the induction of cellular senescence, β-galactosidase activity was assessed. β-Galactosidase-positive cells were significantly more frequent among cells with both PDCD4-specific siRNAs (p2 and k603) than among control and negative control siRNA cells (Fig. 3a, b). PDCD4 knockdown cells showed an altered morphology characterized by a giant cell size with large nuclei in β-galactosidase-positive cells, whereas in control and negative control siRNA-treated cultures, such cells were not observed (Fig. 3a). Taken together, these findings suggested that the cell growth inhibition by PDCD4 knockdown (Fig. 1a) might be due to the induction of senescence.

**Fig. 4:**
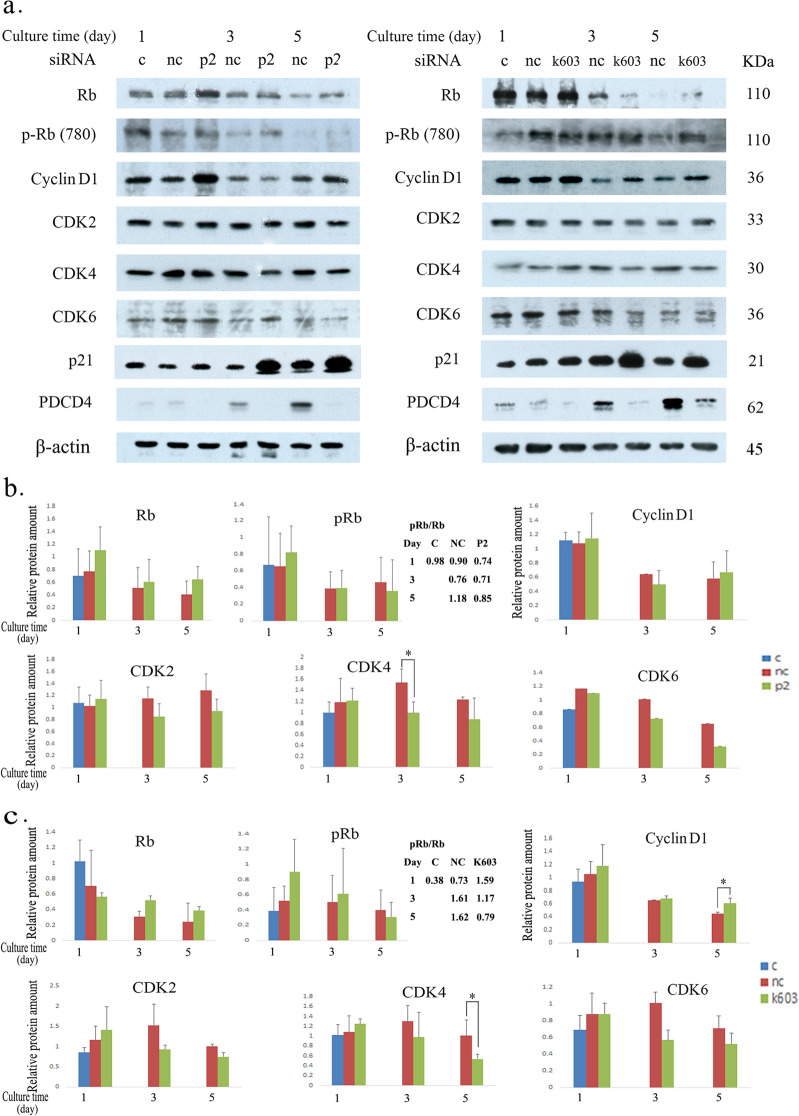
Expression of cell cycle regulatory factors in LX-2 cells. **a** LX-2 cells were treated with p2 PDCD4-specific siRNA (left panel) and k603 PDCD4-specific siRNA (right panel). Protein bands were analyzed by western blotting using antibodies against the respective proteins indicated in **a**. The data represented one of the three experiments. **b** The relative amount of Rb, p-Rb (780) (pRb), cyclin D1, CDK2, CDK4, and CDK6 proteins of p2-specific PDCD4 knockdown cells was determined from **a** (left panel). **c** The relative amount of Rb, p-Rb (780) (pRb), cyclin D1, CDK2, CDK4, and CDK6 proteins of k603-specific PDCD4 knockdown cells was determined from **a** (right panel). The data of **b** and **c** represent the average of three individual experiments. *t* test, *p < 0.05

To elucidate the mechanism underlying cell progression after PDCD4 knockdown, we determined the expression of cell cycle regulatory proteins by western blotting of LX-2 cells after PDCD4 (by p2 and k603 siRNA) knockdown (Fig. 4). The protein level of p21 was upregulated, and that of CDK4 was downregulated significantly, while CDK2 and CDK6 were also downregulated but not significantly. Conversely, cyclin D1 was markedly upregulated in both p2-specific and k603-specific PDCD4 knockdown cells, whereas the upregulation of cyclin D1 in p2 knockdown cells was not significant (Fig. 4b, c). Rb was upregulated in both p2 and k603-siRNA-treated cells compared to control and negative control siRNA-treated cells. Although the levels of phosphorylated Rb were higher than those in negative control cells, the Rb phosphorylation activity might have been suppressed in PDCD4 knockdown cells, given that the p-Rb/Rb ratio of PDCD4 knockdown cells was lower than that of negative control cells (Fig. 4b, c).

### Mechanism underlying the control of p21 expression by PDCD4

**Fig. 5:**
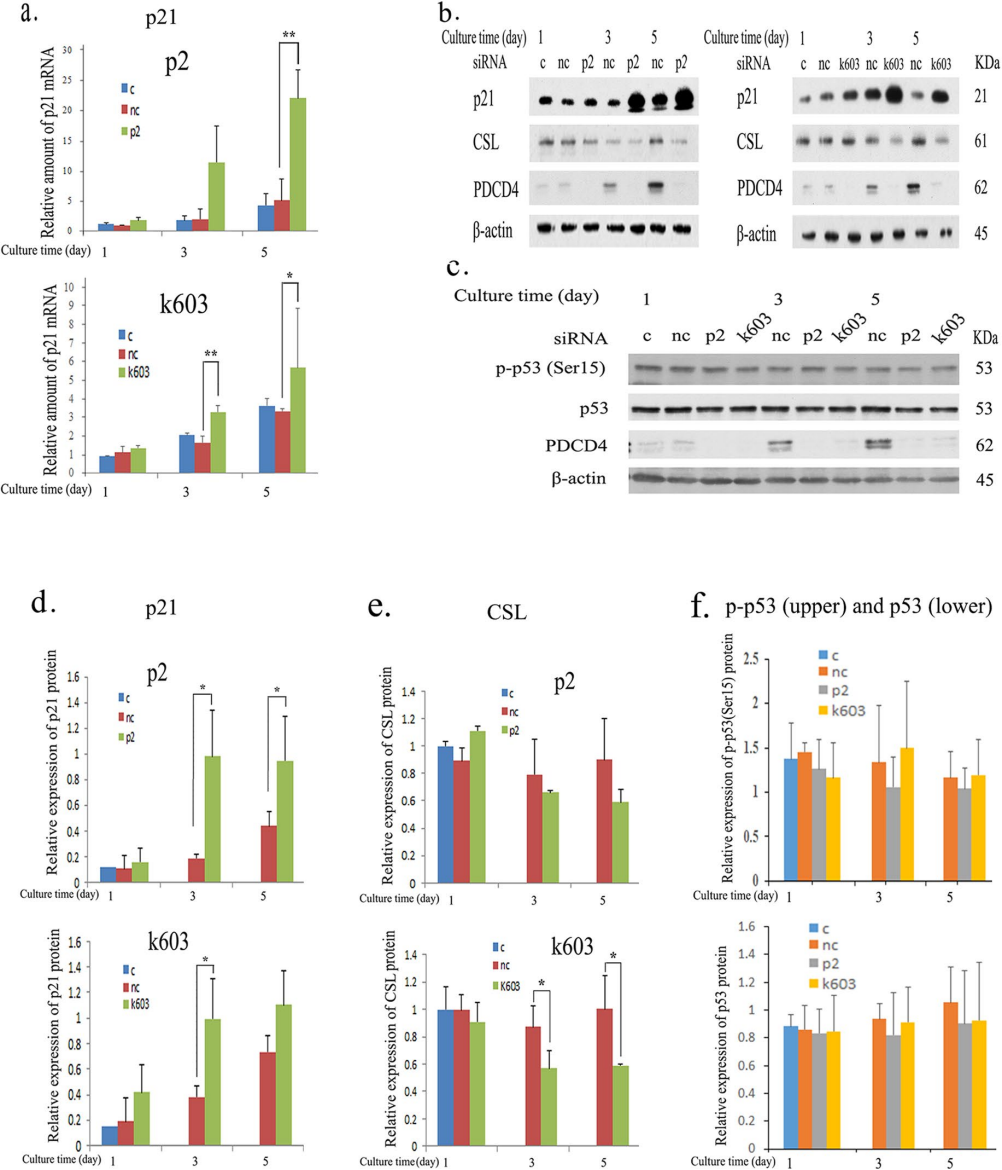
PDCD4 knockdown induced p21 expression in LX-2 cells. **a** LX-2 cells were treated with negative control, p2 (upper panel) and k603 (lower panel) PDCD4-specific siRNAs. The p21 mRNA levels were upregulated by PDCD4 knockdown in LX-2 cells. The data represent the average of three separate experiments. *t* test, *p < 0.05, **p < 0.005. **b** A western blotting assay of PDCD4 knockdown LX-2 cells. LX-2 cells were treated with p2 (left panel) and k603 (right panel) PDCD4-specific siRNAs. Protein bands were analyzed by western blotting using antibodies against the respective proteins indicated in **b**. **c** p-p53 (Ser15) and p53 assay of PDCD4 knockdown LX-2 cells by western blotting. LX-2 cells were treated with p2 and k603 PDCD4-specific siRNAs. Protein bands were analyzed by western blotting using antibodies against the respective proteins indicated in **c**. **d** The relative amount of p21 protein was determined from **b** of LX-2 cells treated with p2 (upper panel) and k603 (lower panel) PDCD4-specific siRNAs. The data represent the average of three separate experiments. *t* test, *p < 0.05. **e** The relative amount of CSL protein determined from **b** of LX-2 cells treated with p2 (upper panel) and k603 (lower panel) PDCD4-specific siRNAs. The data represent the average of three independent experiments. *t* test, *p < 0.05. **f** The relative amount of p-p53 (Ser15) (upper panel) and p53 (lower panel) proteins determined from **c** of LX-2 cells treated with p2 and k603 PDCD4-specific siRNAs

**Fig. 6:**
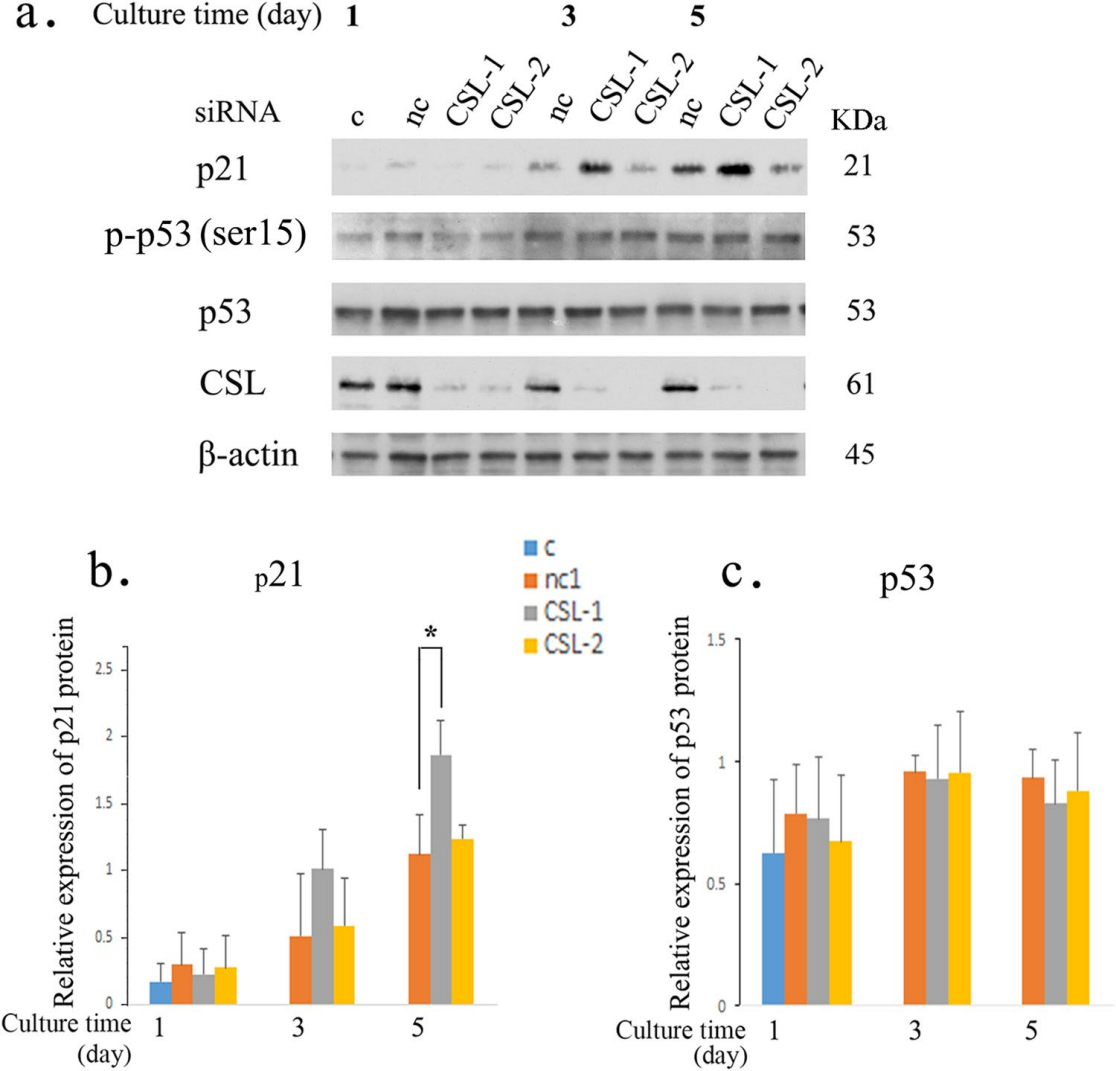
CSL knockdown upregulated the p21 protein level in LX-2 cells. **a** LX-2 cells were treated with CSL-specific siRNA (CSL-1 and CSL-2). Protein bands were analyzed by western blotting using antibodies against the respective proteins indicated in **a**. **b** The relative amount of p21 protein was determined from **a**. **c** The relative amount of p53 was determined from **a**. The data represent the average of three independent experiments. *t* test, *p < 0.05

**Fig. 7:**
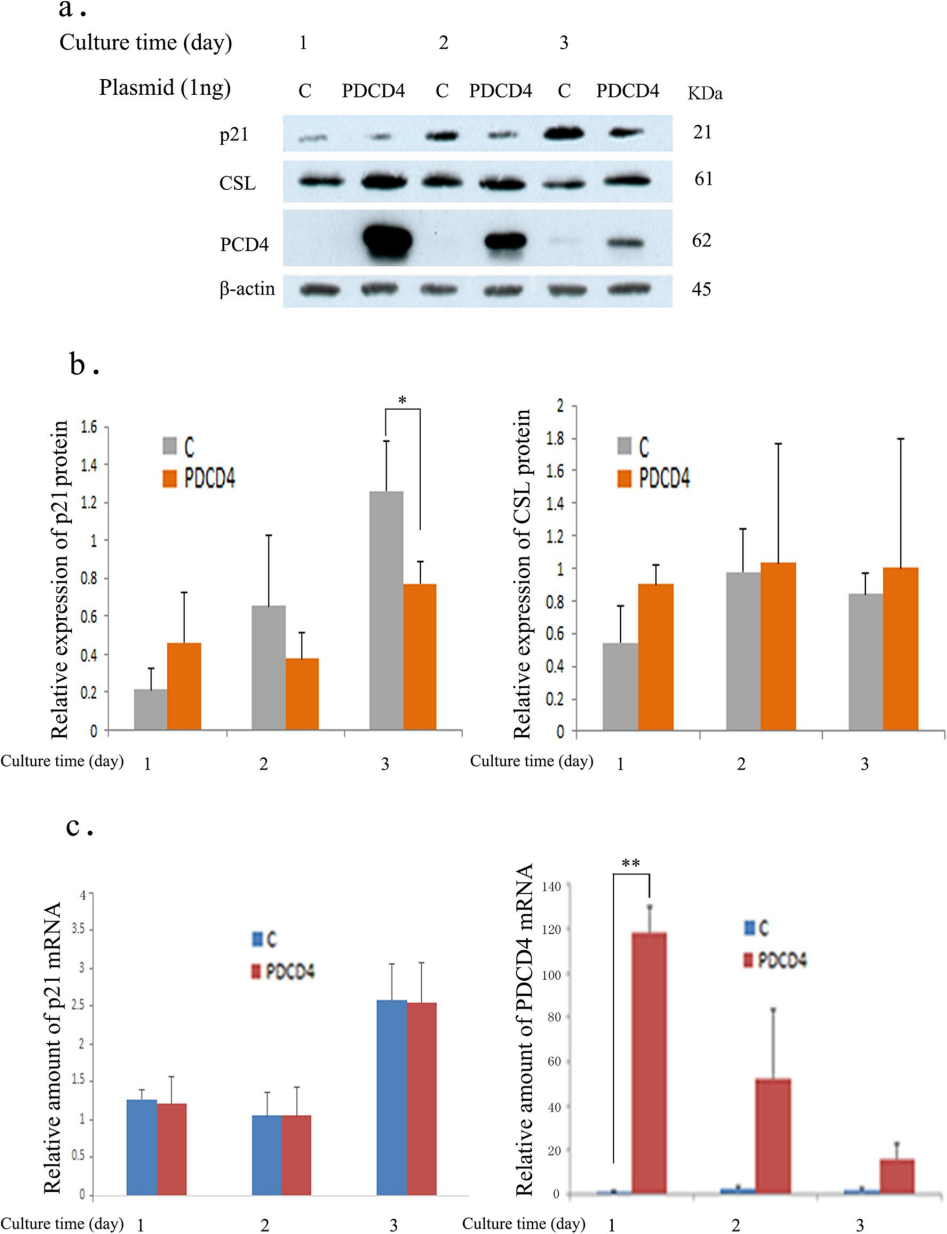
PDCD4 plasmid transfection modulated the expression of p21 and CSL in LX-2 cells. **a** A western blotting assay of PDCD4 plasmid-transfected LX-2 cells. LX-2 cells were transfected with a PDCD4 plasmid (PDCD4) along with a vector plasmid (C). Protein bands were analyzed by western blotting using antibodies against the respective proteins indicated in **a**. **b** The relative amount of p21 (left side) and CSL (right side) proteins was determined from **a**. The data represent the average of three separate experiments. *t* test, *p < 0.05. **c** The relative amount of p21 (left panel) and PDCD4 (right panel) mRNA of LX-2 cells transfected with PDCD4 plasmid (PDCD4) along with vector plasmid (C) was determined by qRT-PCR using the standard curve method. The data represent the average of three individual experiments. *t* test, **p < 0.005

As shown in Fig. 5a, b and d, p21 was upregulated at both the protein and mRNA levels in PDCD4 knockdown cells. Since p21 is a potential target gene of p53, the protein levels of total p53 and phosphorylated p53 at Ser15 that stimulates transcription [35] were also determined, and both were found to be unchanged in PDCD4 knockdown cells (Fig. 5c, f). However, as shown in Fig. 5b, e, PDCD4 knockdown by both kinds of siRNAs (p2 and k603) significantly downregulated the expression of RBPJ-κ/CSL, a downstream notch signaling mediator that acts as a repressor of p21 expression [36]. To determine whether or not the p21 expression is regulated by CSL, LX-2 cells were treated with two different kinds of CSL-specific siRNAs (CSL-1, CSL-2). The protein level of p21 was upregulated significantly by CSL-1 siRNA, but CSL-2 siRNA-mediated upregulation was not effective (Fig. 6a, b), while the levels of p53 were unaffected by either knockdown (Fig. 6a, c). To investigate the p53-independent regulation mechanism of p21 expression, LX-2 cells were transfected with PDCD4 plasmids, and the p21 and CSL protein levels were determined. The overexpression of PDCD4 reduced the protein levels of p21 without changing the CSL expression (Fig. 7a, b). These results collectively suggest that PDCD4 might act as a co-repressor of CSL and regulate the p21 expression in LX-2 cells. After the transfection of the PDCD4 plasmid, the mRNA level of p21 was also determined by qRT-PCR but appeared to be unchanged (Fig. 7c, left panel).

### PDCD4 knockdown upregulates α-SMA in LX-2 cells

**Fig. 8:**
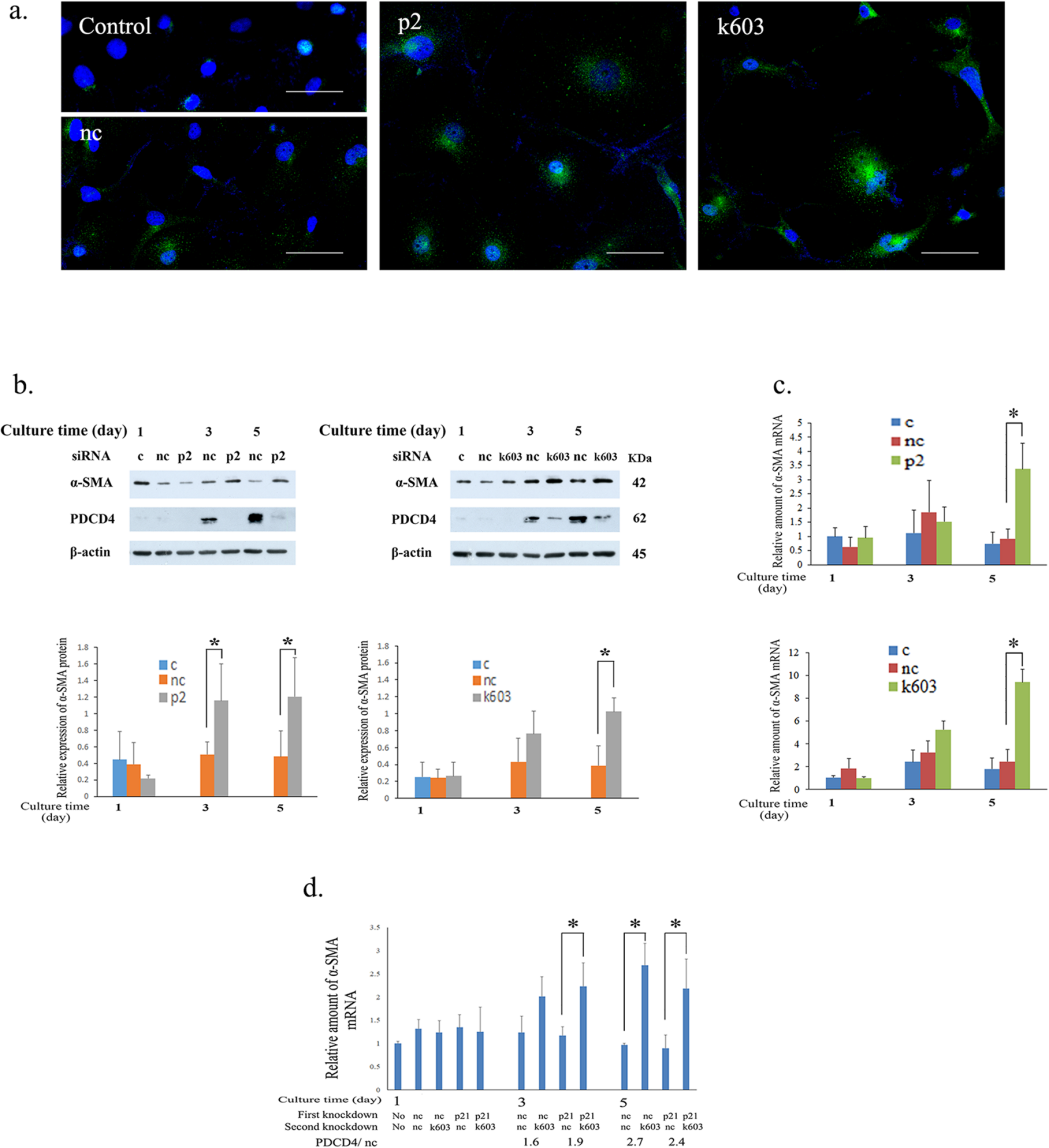
PDCD4 knockdown upregulated the expression of α-SMA in LX-2 cells. **a** Immunofluorescence staining of α-SMA in PDCD4 knockdown cells. Four-day culture after treatment and immunostaining of control (left-upper panel), negative control (left- lower panel), p2 (middle), and k603 (right) PDCD4-specific siRNA-treated LX-2 cells was performed as described in the Materials and methods. Scale bar indicates 50 µm. **b** Western blotting of PDCD4 knockdown LX-2 cells. LX-2 cells were treated with p2 (left panels) and k603 (right panels) PDCD4-specific siRNA and cultured for the indicated times mentioned in **b**. The protein bands were determined by western blotting using antibodies against the respective proteins mentioned in the figure. The upper panels represent the western blot analysis. In the lower panels, the relative protein amounts of α-SMA were determined based on the upper panel of **b**. The data represent the average of three independent experiments. *t* test, *p < 0.05. **c** LX-2 cells were treated with PDCD4-specific siRNAs, similar to in **b**. The α-SMA mRNA levels of PDCD4 knockdown cells were determined by qRT-PCR. The data represent the average of three independent experiments. *t* test, *p < 0.05. **d** p21 knockdown did not rescue the upregulation of α-SMA induced by PDCD4 knockdown. Approximately 1.5 × 10^6^ LX-2 cells were seeded in 100-mm dishes after treatment with negative control siRNA (nc) and p21 siRNA (p21). Twenty-four hours after p21 knockdown, the cells were treated with k603-specific PDCD4 siRNA, and culture was continued for 1, 3, and 5 days. These double-knockdown cells were then collected on the relevant day to detect the mRNA levels. The relative α-SMA mRNA levels of p21 knockdown followed by PDCD4 (k603-specific) siRNA-treated cells were determined. These experiments were repeated twice, and similar results were obtained. The figure represents the data of one experiment

To elucidate the effect of PDCD4 knockdown on LX-2 cell activation, we examined the expression of α-SMA in PDCD4 knockdown LX-2 cells. PDCD4-specific p2 and k603-siRNA-mediated knockdown cells were immunostained with α-SMA antibody. As shown in Fig. 8a, cells with a high expression of α-SMA were observed in both p2 and k603-specific PDCD4 siRNA treatment compared to control and negative control cells. The protein levels of α-SMA were upregulated after 3 days in both p2 and k603-specific PDCD4 siRNA knockdown cells (Fig. 8b). Significant differences in protein levels were observed at days 3 and 5 in p2, and at day 5 in k603-siRNA-treated LX-2 cells (Fig. 8b, lower panel). The mRNA levels were also upregulated by both kinds of siRNAs (Fig. 8c). To examine whether or not the upregulation of α-SMA after PDCD4 knockdown is p21 dependent, we next treated cells with p21 siRNA. As shown in Fig. 8d, p21 knockdown failed to reverse the condition of the α-SMA mRNA levels of PDCD4 knockdown cells, suggesting that upregulation of α-SMA was p21 independent.

**Fig. 9:**
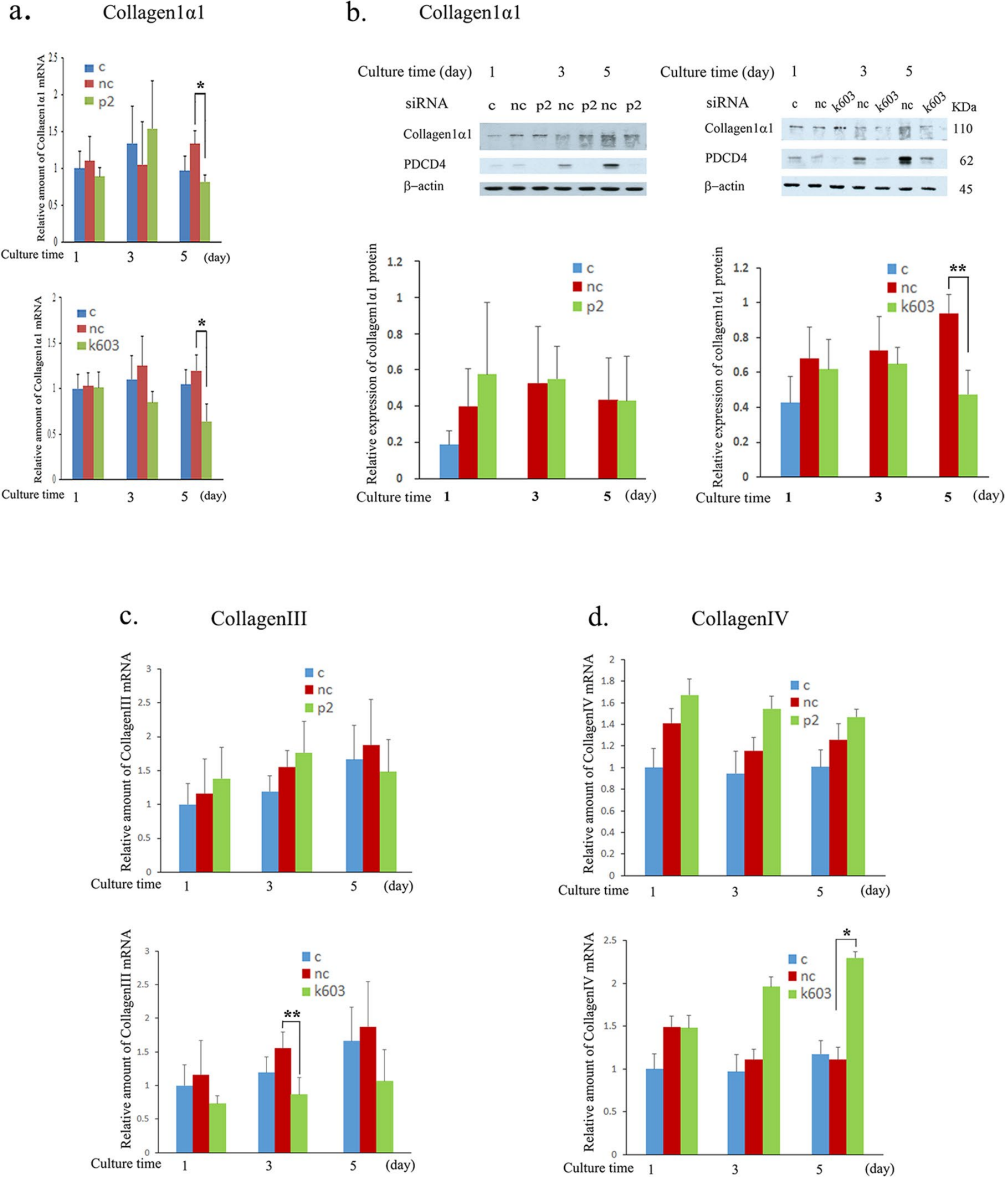
PDCD4 knockdown downregulated the collagen1α1 and collagen III mRNA levels but upregulated collagen IV levels in LX-2 cells. LX-2 cells were treated with p2 and k603 PDCD4-specific siRNAs. The mRNA and protein levels of the knockdown cells were determined as described in the Materials and methods. **a** The relative amount of collagen1α1 mRNA of p2 (upper panel) and k603 (lower panel) PDCD4-specific siRNA-treated LX-2 cells. The collagen1α1 expression was significantly downregulated by both siRNA treatments at day 5. **b** A western blotting analysis of collagen1α1 of PDCD4 knockdown LX-2 cells. LX-2 cells were treated with p2 (left panels) and k603 (right panels) PDCD4-specific siRNA and cultured for the indicated times mentioned in **b**. Upper panel, the protein bands were analyzed by western blotting using antibodies against the respective proteins mentioned in the figure. Lower panel, the amount of collagen1α1 protein levels obtained from the upper panel. Collagen1α1 protein levels were significantly downregulated in k603 PDCD4-specific siRNA-treated cells (right site) compared to negative control cells but not in p2 PDCD4-specific siRNA-treated cells (left site). The data represent the average of three separate experiments. **c** The relative amount of collagen III mRNA of p2 (upper panel) and k603 (lower panel) PDCD4-specific siRNA-treated LX-2 cells. The collagen III mRNA expression was downregulated by both siRNA treatments. **d** The relative amount of collagen IV mRNA of p2 (upper panel) and k603 (lower panel) PDCD4-specific siRNA-treated LX-2 cells. The collagen IV mRNA expression was upregulated by PDCD4-specific siRNA treatments. *t* test, *p < 0.05, **p < 0.005

### PDCD4 knockdown downregulates the collagen1α1 and collagen III genes but upregulates the collagen IV and MMP genes

**Fig. 10:**
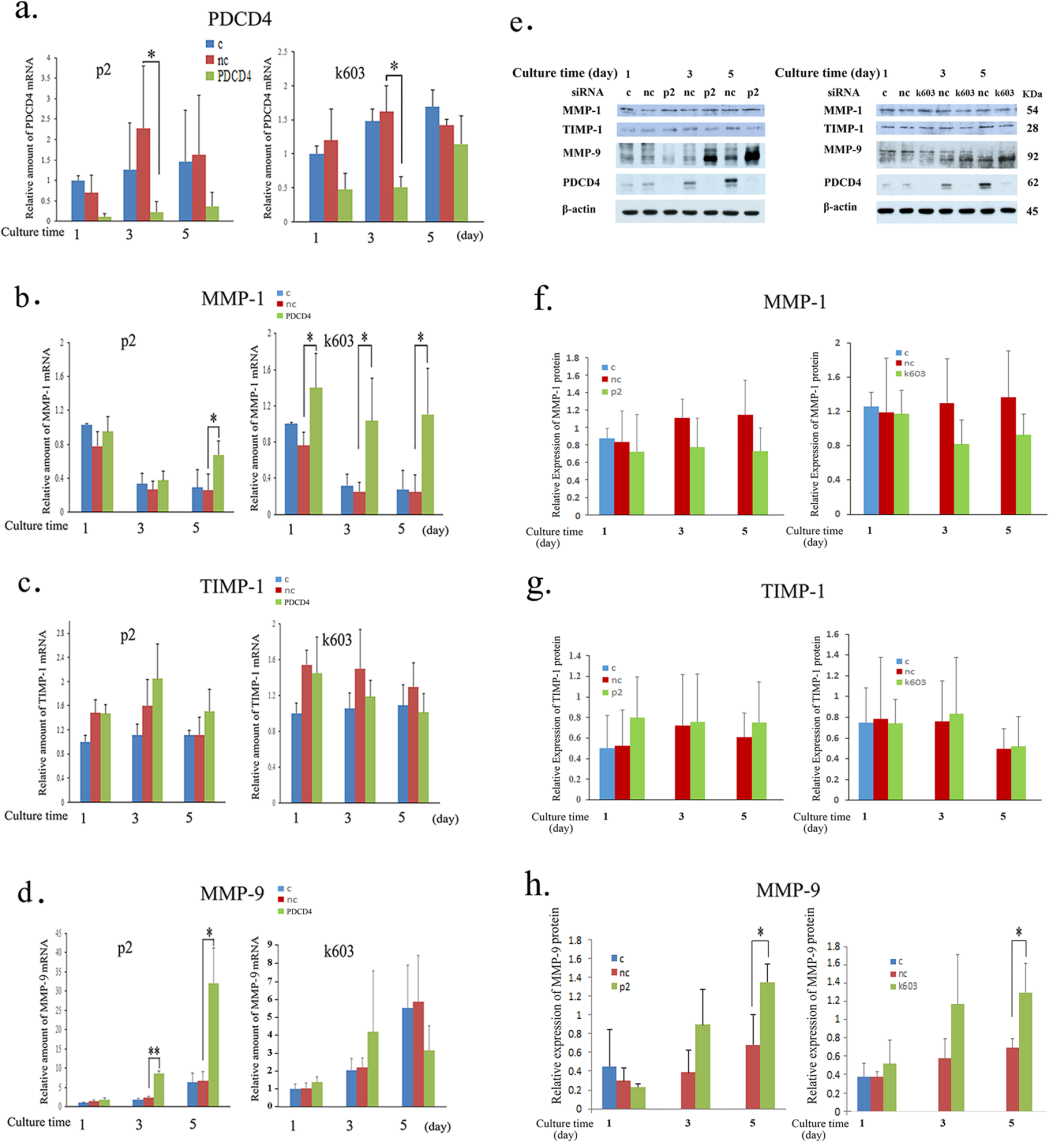
PDCD4 knockdown upregulated the mRNA levels of MMP-1 and MMP-9 and the protein levels of MMP-9 in LX-2 cells. LX-2 cells were treated with p2 and k603 PDCD4-specific siRNAs. **a** The relative amount of PDCD4 mRNA of p2 (left panel) and k603 (right panel) PDCD4-specific siRNA-treated LX-2 cells. **b** The relative amount of MMP-1 mRNA of p2 (left panel) and k603 (right panel) PDCD4-specific siRNA-treated LX-2 cells. The MMP-1 expression was significantly upregulated by both siRNA treatments. **c** The relative amount of TIMP-1 mRNA of p2 (left panel) and k603 (right panel) PDCD4-specific siRNA-treated LX-2 cells. **d** The relative amount of MMP-9 mRNA of p2 (left panel) and k603 (right panel) PDCD4-specific siRNA-treated LX-2 cells. In the case of k603-mediated knockdown, while the mRNA level of MMP-9 was consistently upregulated at 1 and 3 days, no statistical significance was noted due to the large differences between cultures. **e** A western blot analysis of PDCD4 knockdown LX-2 cells. LX-2 cells were treated with p2 (left panels) and k603 (right panels) PDCD4-specific siRNAs and cultured for the indicated times mentioned in **e**. The protein bands were analyzed by western blotting using antibodies against the respective proteins mentioned in the figure. **f** The relative amount of MMP-1 protein was determined from **e** of LX-2 cells treated with p2 (left panel) and k603 (right panel) PDCD4-specific siRNAs. The data represent the average of three separate experiments. **g** The relative amount of TIMP-1 protein was determined from **e** of LX-2 cells treated with p2 (left panel) and k603 (right panel) PDCD4-specific siRNAs. The data represent the average of three separate experiments. **h** The relative amount of MMP-9 protein was determined from **e** of LX-2 cells treated with p2 (left panel) and k603 (right panel) PDCD4-specific siRNAs. The data represent the average of three separate experiments. *t* test, *p < 0.05, **p < 0.005

**Fig. 11:**
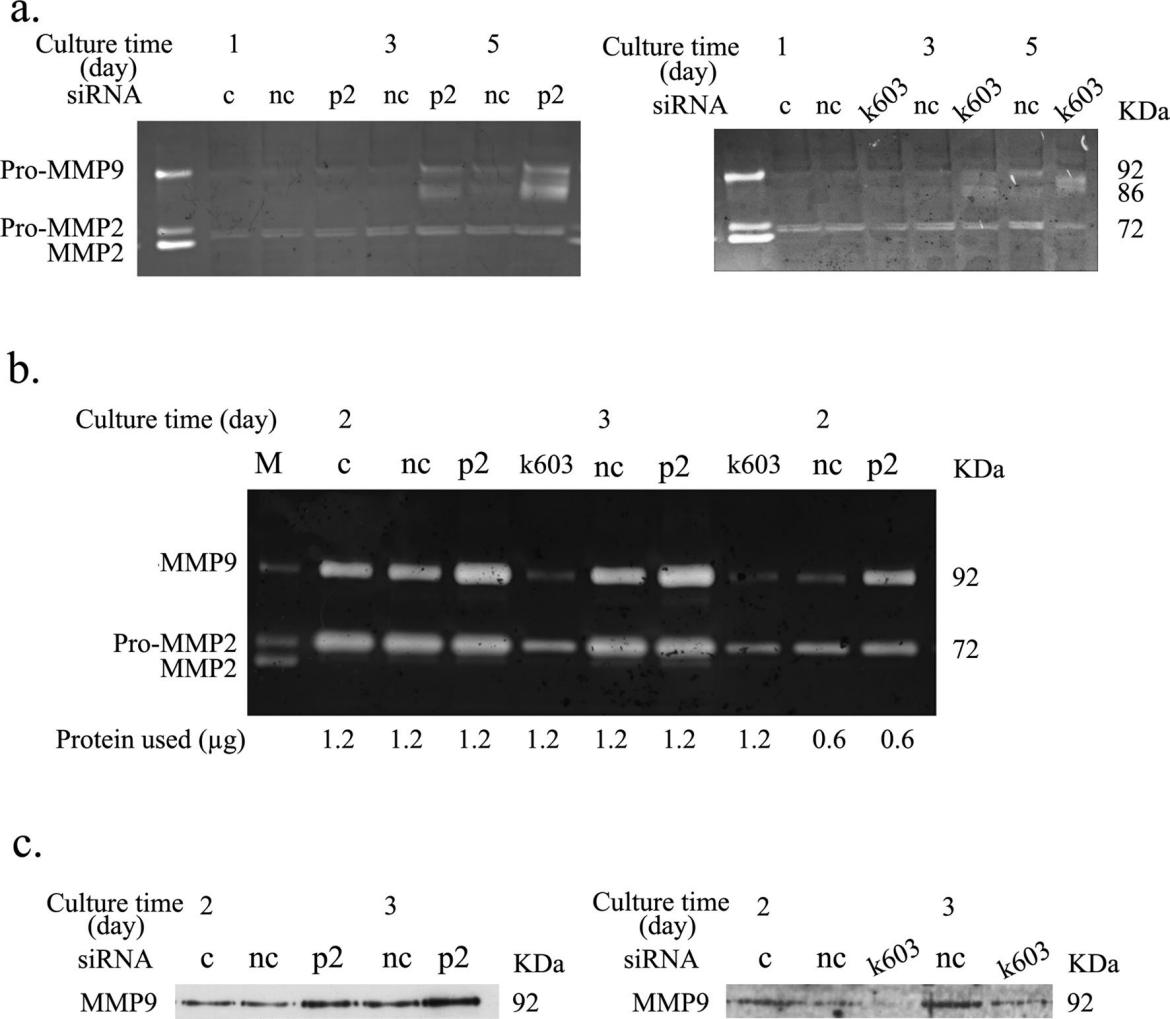
PDCD4 knockdown upregulated the proteolytic activity of MMP-9 in LX-2 cells. **a** Gelatin zymography of PDCD4 knockdown LX-2 cell extract. LX-2 cells were treated with p2 (left panels) and k603 (right panels) PDCD4-specific siRNA and cultured for the indicated times mentioned in **a** and were subjected to gelatin zymography. Pro-MMP-9 and active MMP-9 was upregulated by both kinds of siRNA-treated cells. **b** Gelatin zymography of serum-free conditioned medium of PDCD4 knockdown LX-2 cells. LX-2 cells were treated with p2 and k603 PDCD4-specific siRNAs, and the conditioned medium was collected as mentioned in the Materials and methods. The amount of protein indicated in **b** was subjected to gelatin zymography. Proteolytic activity of MMP-9 was observed at 92 kDa. **c** Western blotting of the serum-free conditioned medium of p2 (left panel) and k603 (right panel) PDCD4-specific siRNA-treated cells. Approximately 3 µg of protein of the conditioned medium used in **b** was used as input for western blotting

To evaluate the impact of the PDCD4 function on ECM regulation, mRNA levels of collagen1α1, collagen III, and collagen IV were determined in PDCD4 knockdown LX-2 cells. Collagen1α1 and collagen III mRNA levels were downregulated, but collagen IV mRNA levels were upregulated in these cells (Fig. 9a, c and d). Collagen1α1 protein levels were also measured and found to be downregulated in k603 siRNA-mediated knockdown cells; however, in p2 siRNA-treated cells, the protein levels were not altered compared to negative control siRNA-treated cells (Fig. 9b). MMP-1 and MMP-9 as well as TIMP-1 levels were determined in PDCD4 knockdown LX-2 cells (Fig. 10). PDCD4 knockdown upregulated the mRNA levels of MMP-1 but downregulated the expression of the protein (Fig. 10b, e and f). Both the mRNA and proteins levels of TIMP-1 were unchanged in PDCD4 knockdown cells (Fig. 10c, e and g). In contrast, both the mRNA and protein levels of MMP-9 were upregulated in PDCD4 knockdown cells (Fig. 10d, e and h). The proteolytic activity of MMP-9 in p2-specific PDCD4 knockdown cell extracts was prominently upregulated compared to negative control treated cells at 3 and 5 days (Fig. 11a-left panel), whereas in k603-specific PDCD4 knockdown cell extracts, the upregulation of proteolytic activity was reduced (Fig. 11a-right panel). In the conditioned medium of PDCD4 knockdown cells, the proteolytic activity of MMP-9 was upregulated in p2-specific siRNA-treated cells but downregulated in k603-specific siRNA-treated cells compared to negative control and control cells (Fig. 11b). Western blotting of conditioned media showed that the protein level of MMP-9 was upregulated in p2-specific siRNA-treated cells (Fig. 11c, left panel) but downregulated in k603-specific siRNA-treated cells (Fig. 11c, right panel). In the case of k603 siRNA-mediated knockdown, different culture conditions were applied, but no upregulation of MMP-9 was observed (data not shown).

**Fig. 12:**
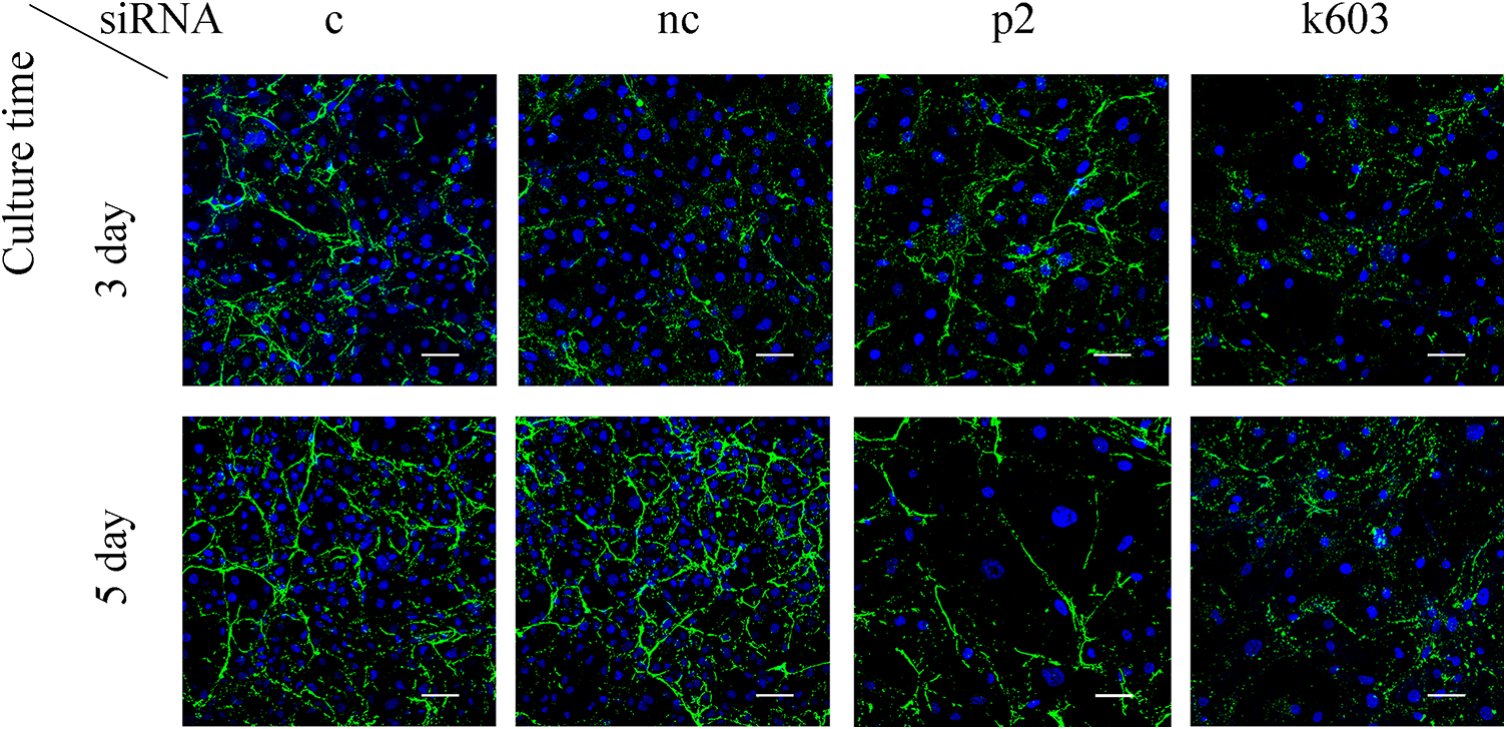
PDCD4 knockdown modulated the immunocytochemical staining pattern of collagen1α1. LX-2 cells were treated with negative control, p2, and k603-PDCD4-specific siRNAs. At days 3 and 5 after knockdown, cells were immunocytochemically stained with collagen1α1 antibody as described in the Materials and methods. Scale bar indicates 50 µm

Finally, the collagen1α1 expression of PDCD4 knockdown cells was immunocytochemically examined. The collagen1α1 expression was decreased in k603-specific siRNA-mediated PDCD4 knockdown cells but increased in p2-specific siRNA-mediated knockdown cells (Fig. 12). In the k603-siRNA-mediated PDCD4 knockdown cells, collagen fibers were reduced, and the rate of a dotted staining pattern was higher than in negative control cells (Fig. 12). In contrast, the collagen1α1 expression was increased at day 3, and fibers were maintained along with dotted staining in the p2-siRNA-mediated PDCD4 knockdown cells compared to negative control cells (Fig. 12). These results were consistent with those for collagen1α1 protein levels, where k603-siRNA-mediated PDCD4 knockdown downregulated the collagen1α1 protein level whereas p2-siRNA-mediated PDCD4 knockdown did not significantly change the level (Fig. 9b).

This finding might indicate that because activation and suppression were induced simultaneously in this PDCD4 knockdown system, for p2-specific siRNA-mediated knockdown, the suppression may be overcome by activation, while in k603-siRNA-mediated PDCD4 knockdown, the activation may be overcome by suppression.

## Discussion

We recently showed that PDCD4 knockdown induced cellular senescence by upregulating the p21 expression and downregulated the cell cycle regulators CDKs in Huh7 hepatoma cells [27]. In the case of LX-2 cells, although PDCD4 knockdown induced premature senescence by upregulating the p21 expression, the suppression of cell proliferation activity was incomplete, and the growth suppression of cells was likely modulated because of the upregulation of cyclin D1 and incomplete downregulation of CDKs after PDCD4 knockdown in LX-2 cells. Guo et al. reported that PDCD4 suppression stimulated cell growth by upregulating the cyclin D1 expression [37]. Regarding the mechanism underlying the control of p21 expression, it was shown that p53-dependent and p53-independent mechanisms were involved in Huh7 hepatoma cells [27, 38]. The binding site of CSL, a specific Notch signaling factor, is located at the − 476 position upstream of the p53 binding site in the p21 promoter region and acts as a repressor in the absence of Notch signaling [36, 39]. Jo et al. reported that PDCD4 is associated with CSL and functions as an inhibitor of gene expression [26]. PDCD4 knockdown did not upregulate the expression of p53 or phosphorylation of p53 at Ser15, which stimulate transcription, but did downregulate the CSL protein levels, so PDCD4 may control the expression of CSL. The CSL system might be a p53-independent control mechanism for the p21 expression in LX-2 cells. While we showed that PDCD4 knockdown upregulated p21 protein levels, PDCD4 overexpression induced the downregulation of p21 protein levels (mRNA levels were unchanged), suggesting that PDCD4 might control p21 expression post-transcriptionally.

Chronic p53-independent p21 expression reportedly caused genomic instability by deregulating replicative licensing genes [40]. In this context, the p53 checkpoint works to limit re-replication by eliminating re-replicating cells through apoptosis with functional p53 [41], although re-replicating cells accumulate and eventually lead to genomic instability in cells with inactive p53. These results suggest that DNA damage by genomic instability may occur in PDCD4 knockdown cells on the inactivation of p53.

Cellular senescence is induced by many factors that propagate various signaling pathways, such as the oncogenic Ras-MAPK pathway and mammalian target of rapamycin (mTOR) pathway [15, 42, 43]. mTORC1 has been shown to induce a senescence-associated phenotype, including the senescence-associated secretory phenotype (SASP) [44, 45]. Active mTORC1 drives protein translation and lipid and nucleotide synthesis to stimulate cells and metabolism [46]. PDCD4 is also known to be a translation inhibitor through competition with eIF4A-containing translation-initiation complex and might act to balance mTORC-stimulated signaling. PDCD4 was conversely downregulated when mTOR was activated in the activation of proteasomal degradation system [47, 48]. Therefore, the downregulation of PDCD4 by siRNA treatment might enhance mTOR-induced senescence.

LX-2 cells were obtained by immortalization of human HSCs, which harbor characteristics of myofibroblasts [49]. Our data indicate that PDCD4 knockdown upregulates the expression of α-SMA, which is used as activation marker of HSCs. Generally, activation of HSCs leads to increased production of ECM [2], including collagen1α1; however, PDCD4 knockdown in LX-2 cells showed the downregulation of collagen1α1 and collagen III expression and the upregulation of MMP-1 and MMP-9 expression, which might facilitate ECM degradation and promote fibrosis resolution. A similar phenotype of activated but senescent HSCs has been observed in non-alcoholic steatohepatitis (NASH)-HCC animal models, demonstrating HCC development without significant liver fibrosis [50]. Therefore, PDCD4 may help limit the development of liver fibrosis.

However, the mechanisms underlying the control of PDCD4 for regulating the expression of α-SMA, collagen family, MMP-1, MMP-9, and TIMP-1 in LX-2 cells are still unclear. Our results showed that changes in the fibrogenic phenotype of PDCD4 knockdown LX-2 cells were not p21 dependent, suggesting that the profibrogenic phenotype might be regulated by cell growth-independent mechanisms regulated by PDCD4. Snail, a zinc finger transcription factor, increases cancer invasion by upregulating members of the MMP family, such as MMP-1, MMP-2, and MMP-7, in hepatocellular carcinoma [51]. It was also reported that Snail-specific siRNA treatment reduced the invasive ability in Panc-1 and IMR-90 cells [52]. Wang et al. reported that PDCD4 knockdown upregulated the expression of Snail followed by upregulation of Sin1 protein levels [53]. In HepG2 cells, downregulation of NF-κB led to a reduction in the expression of MMP-9 and subsequently reduced invasion [54]. PDCD4 reportedly inhibits NF-κB-mediated transcription by directly interacting with p65, a component of NF-κB [55]. It has also been reported that MMPs contain an AP-1 binding site in the promoter regions of all MMP genes [56]. Therefore, apart from direct regulation of the transcription of targeted genes, PDCD4 may regulate transcription via an indirect mechanism, as PDCD4 knockdown upregulates AP-1 activity [57].

In conclusion, PDCD4 knockdown induced premature senescence in human HSCs and changed their phenotype to a less-fibrogenic one, suggesting that PDCD4 may be a target for limiting liver fibrosis.

## Data Availability

All data, generated and analyzed during this study, supporting the conclusions of this article are included within the article.
